# Composition-Dependent Dielectric Properties of DMF-Water Mixtures by Molecular Dynamics Simulations

**DOI:** 10.3390/ijms10041590

**Published:** 2009-04-14

**Authors:** Guo-Zhu Jia, Ka-Ma Huang, Li-Jun Yang, Xiao-Qing Yang

**Affiliations:** College of Electronics and Information Engineering, Sichuan University, Chengdu, 610064, P.R. China; E-Mails: jia1689500@126.com (G.-Z.J.); yyxxqq_mail@163.com (X.-Q.Y.); ljyang320@163.com (L.-J.Y.)

**Keywords:** DMF-H_2_O, Dielectric Properties, Mixture, Molecular Dynamics

## Abstract

In this paper, we study the dielectric properties of water-*N,N* dimethylformamide (DMF) mixtures over the whole composition range using a molecular dynamics (MD) simulation. The static and microwave frequency-dependent dielectric properties of the mixtures are calculated from MD trajectories of at least 2 ns length and compared to those of available measurements. We find that the short-ranged structural correlation between neighboring water and DMF molecules strongly influences the static dielectric properties of mixtures. In terms of the dynamics, we report time correlation functions for the dipole densities of mixtures and find that their long-time behavior can be reasonably described by biexponential decays, which means the dielectric relaxations of these mixtures are governed by complex multitimescale mechanisms of rotational diffusion. The dipole density relaxation time is a non-monotonic function of composition passing through a maximum around 0.5 mole fraction DMF, in agreement with the measured main dielectric relaxation time of mixtures.

## 1. Introduction

It is well known that microwaves can speed up chemical reactions for the synthesis of organic and inorganic materials [1,2], and sinter metal oxides with high-energy efficiency [3]. The physical and chemical properties of matter are the basis of materials science, engineering, and biological applications. Because DMF-aqueous solutions are a simple biochemical model of biological molecular aqueous solutions [4], examination of aqueous DMF solutions may be a useful tool for studying the local electrical and biological properties of samples exposed to microwave frequency. The amides represent an important class of organic solvents due to their high polarity, strong solvating power, and a large liquid state range [5–9]. Substitution at the amide nitrogen atom allows control of the extent of intermolecular hydrogen bonding, which is dominating factor for the physical properties exhibited by the liquids. Besides, amides may be used model systems for peptides. A typical example is DMF [10]. Although some investigations have been devoted to the molecular dynamics of the DMF [11], the influence of hydrogen bonding on its aqueous solutions is not well understood. The aim of this contribution is to address these questions by studying the dielectric relaxation behavior of DMF aqueous solutions at room temperature because dielectric properties are fundamental for a deep understanding of polar liquids [12–17]. Computer simulations provide a suitable tool to analyze these properties at molecular level, providing a direct route from microscopic details to macroscopic properties of experimental interest. To study the dielectric properties of DMF aqueous solutions, we have performed MD simulations. An OPLS all-atom force field is used for the simulation of DMF, revealing the local order and formation of the weak hydrogen bond of C–H…O, and TIP5P adopted for the simulation of water [11] was used to describe intermolecular interaction potentials. The MD simulation method is suitable for this purpose since it enables one to get both static and microwave frequency-dependent dielectric properties. However, difficulties arise due to the slow relaxation of the time correlation functions of interest and to the sensitivity to the long-range intermolecular interactions of the quantities involved. To overcome the first difficulty, extremely long simulations should be performed whereas large systems are required to overcome the latter [18–20]. Moreover, statistically accurate calculations of these collective properties will also need very long simulations since only one value of a given collective quantity is got at each time step.

## 2. Methodology

### 2.1. Force fielids and simulation details

Simple rigid potential models were used for both DMF and water. The nonbonded interactions are represented by a sum of the Coulomb and Lennard-Jones terms when Equation 1 and Lorentz-Berthelot combining rules are used [11]:

(1) $$E_{ab}\;=\;\left\{\sum_{i}^{a}\sum_{j}^{b}\frac{q_{i}q_{j}}{r_{ij}}+4{ɛ}_{LJ}\;\left[{\left(\frac{\sigma }{r_{ij}}\right)}^{12}-{\left(\frac{\sigma }{r_{ij}}\right)}^{6}\right]\right\},\;\sigma \;=\;\frac{1}{2}(\sigma_{i}+\sigma_{j}),\;{ɛ}_{LJ}=\sqrt{{ɛ}_{i}{ɛ}_{j}}$$

where *E*_ab_ is the interaction energy between molecules a and b. For water molecules, the TIP5P model is adopted. TIP5P is a new five-site, nonpolarizable water model that was fitted by Mahoney and Jorgensen on the pure liquid water properties by MC simulation and reproduces the density of liquid water accurately over a large temperature range. OPLS-AA (optimized potentials for liquid simulations-all atom) was recently developed for the all-atom force field. For the DMF molecule, a modified OPLS-AA model is adopted in bracket. *N*-methyl group parameters agree well with experimental values of the density and heat of vaporization so that OPLS-AA work will be hardly affected. Table 1 lists the potential parameters for the pure components.

Our simulations are run in the NVE ensemble consisting of molecules placed in a cubic box with periodic boundary conditions at an average temperature of 298 K and a simulation number of molecules between 82 and 216. The box length is chosen to match the experimental density of DMF at 298 K and 1 atm. The Lennard-Jones interactions are cutoff at 1/2 box length and the long-ranged portions of the electrostatic potentials are treated by Ewald sums with conducting boundaries. [20] The motion equations are integrated using the leap-frog algorithm [21] with a time step of 2.5 fs, while the molecular geometries are restored using SHAKE. [22] The results reported here are based on averages over about 800,000 MD time steps (2 ns) for each model. The model simulation parameters of mixtures are listed in Table 2.

## 3. Results and Discussion

### 3.1. Theoretical framework

For an infinite system with cubic symmetry, the dielectric permittivity and susceptibility tensors are related through [19–23]:

(2) $$\frac{{ɛ}_{L}(k,\;\omega )\;-\;{ɛ}_{\infty }}{{ɛ}_{L}(k,\;\omega ){ɛ}_{\infty }}=\;\chi_{L}^{0}\left(k,\;\omega \right),\;{ɛ}_{T}\left(k,\;\omega \right)\;-\;{ɛ}_{\infty }\;=\;\chi_{T}^{0}\left(k,\;\omega \right)$$

for the longitudinal and transverse components, respectively. *ɛ*_∞_ is the dielectric constant at optical frequencies (for a model system with nonpolarizable molecules, *ɛ*_∞_ =1). Linear response theory relates the susceptibility tensor *χ*^0^(*k*,*ω*) to correlation functions of the Fourier components of the dipole density of the system $\vec{M}\left(\vec{k},\;t\right)$ The two components of $\vec{M}\left(\vec{k},\;t\right)$:

(3) $$\vec{M}\left(\vec{k},\;t\right)\;=\;{\vec{M}}_{L}\left(\vec{k},\;t\right)\;+\;{\vec{M}}_{T}\left(\vec{k},\;t\right)$$

can be expressed as follows:

(4) $${\vec{M}}_{L}\left(\vec{k},\;t\right)\;=\;\sum_{j=1}^{N}\hat{k}\hat{k}\cdot {\vec{\mu }}_{j}(t)\;\text{exp}\left(i\hat{k}{\vec{r}}^{CM}(t)\right)$$

(5) $${\vec{M}}_{T}\left(\vec{k},\;t\right)\;=\;\sum_{j=1}^{N}\left(1\;-\;\hat{k}\hat{k}\right)\cdot {\vec{\mu }}_{j}\left(t\right)\text{exp}\left(i\hat{k}{\vec{r}}^{CM}\left(t\right)\right)$$

where ${\vec{\mu }}_{j}\left(t\right)$ is the dipole moment of the *j*th molecule and ${\vec{r}}^{CM}\left(t\right)$ the position of its center-of-mass. The components of the susceptibility tensor are given by [14–15]:

(6) $$\chi_{A}^{0}\left(k,\;\omega \right)\;=\;\frac{\langle {\left|{\vec{M}}_{A}\left(\vec{k},\;0\right)\right|}^{2}\rangle }{\upsilon_{A}Vk_{B}T{ɛ}_{0}}[1\;+\;i\omega \Phi_{A}\left(k,\;\omega \right)]$$

where A = L or T(υ*_L_* = 1, υ*_T_* = 2), *V* is the volume of the sample, ɛ_0_ is the vacuum permittivity, and Φ*_A_* (*k*,ω) is the Fourier transform:

(7) $$\Phi_{A}\left(k,\;\omega \right)\;=\;\int_{0}^{\infty }dt\Phi_{A}\left(k,\;t\right)\;\text{exp}(i\omega t)$$

of the normalized dipole density correlation function: Φ*_A_* (*k,t*) defined by:

(8) $$\Phi_{A}\left(k,\;t\right)\;=\;\frac{\langle {\vec{M}}_{A}\left(\vec{k},\;t\right)\cdot {\vec{M}}_{A}\left(-\vec{k},\;0\right)\rangle }{\langle {\left|{\vec{M}}_{A}\left(\vec{k},\;0\right)\right|}^{2}\rangle }$$

Because of the short-ranged nature of the correlations which determine the dielectric permittivity, ɛ (*k*,ω) must become independent of the wave-vector number when *k* is sufficiently small, that is:

(9) $$\underset{k\to 0}{\text{lim}}{ɛ}_{L}(k,\;\omega )\;=\;\underset{k\to 0}{\text{lim}}{ɛ}_{T}\left(k,\;\omega \right)\;=\;ɛ\left(\omega \right)$$

In the low *k* limit the following condition must be fulfilled:

(10) $$\underset{k\to 0}{\text{lim}}{ɛ}_{L}(k)\;=\;\underset{k\to 0}{\text{lim}}{ɛ}_{T}\left(k\right)\;=\;{ɛ}_{0}$$

Equations (9) – (10) provide a route to determine ɛ (ω) and ɛ_0_, respectively. However, due to the periodic boundary conditions used during the computer simulations, the $\vec{k}$ vectors which may be studied are restricted to: $\vec{k}\;=\;\frac{2\pi }{L}\left(l,m,n\right)$, where *l, m, n* are integers and *L* is the length of the cubic box. Thus, the smallest wave-vector which may be studied is ${\text{k}}_{\text{min}}=\frac{2\pi }{L}$. Whether the low *k* limit is reached can be tested through the consistency of the calculated ɛ*_L_* (*k*_min_, ω) and ɛ*_T_* (*k*_min_, ω). Another approach for the determination of ɛ (ω) is based on the calculation of the total dipole moment of the primitive cube of the simulation $\vec{M}\left(t\right)\;=\;\sum_{j=1}^{N}{\vec{\mu }}_{j}\left(t\right)$. If the Ewald summation technique is used to handle with the long range interactions and conducting walls boundary conditions are assumed, the relation between ɛ (ω) and the total dipole moment correlation is given by [24–25]:

(11) $$ɛ\left(\omega \right)-{ɛ}_{\infty }\;=\;\frac{\langle {\left|\vec{M}\left(0\right)\right|}^{2}\rangle }{3Vk_{B}T{ɛ}_{0}}[1+i\omega \Phi \left(\omega \right)]$$

where Φ(ω) is the Fourier transform of the $\vec{M}\left(t\right)$ autocorrelation function:

(12) $$\Phi \left(t\right)\;=\;\frac{\langle \vec{M}\left(t\right)\cdot \vec{M}\left(0\right)\rangle }{\langle {\left|\vec{M}\left(0\right)\right|}^{2}\rangle }$$

In the classical limit (*ħ* = *h* / 2π → 0, *h* is the Planck constant), from Equations (11) – (12) has obtained the following form:

(13) $${ɛ}^{\prime \prime }\;=\;\left({ɛ}_{0}-{ɛ}_{\infty }\right)\omega \int_{0}^{\infty }dt\Phi \left(t\right)\text{cos}\left(\omega t\right)$$

**Figure 1.**
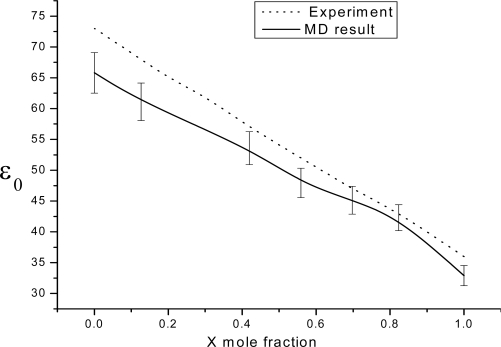
MD (full symbols and solid line), and experiment results [19] (doted line), for the mixture’s dielectric constant vs DMF mole fraction. The solid lines are drawn as guides to the eye.

We begin our analysis by presenting the results for the dielectric constant ɛ_0_ as a function of the composition, which is depicted by solid symbols in Figure 1, along with the experimental values from the Luzar theory results. The MD data shown have been evaluated from the **k** = 0 correlations (equation 11 at ω = 0), and the error bars are estimated using the blocking method of Flyvbjerg and Petersen[26]. Considering that the the MD simulations are long enough to reach a plateau value for ɛ_0_ and that this plateau value is quite sensitive to the initial conditions, the MD values of ɛ_0_ for the mixtures as well as the trend with composition compare fairly well with the Luzar theory results from ref 19 (doted line) with the maximum number of DMF-water H-bonds fixed at 0.419. Despite its simplicity, the Luzar theory treatment reveals the importance of H-bonding to the dielectric constant of this mixture. Above 0.7 DMF, the agreement between the Luzar theory and simulated dielectric constants seems to be somewhat better than for other compositions. It is interesting to notice that good agreement is also found for the average number of DMF-water and water-water H-bonds predicted by the theory and the MD simulations [11] around these compositions.

## 4. Dynamic Dielectric Properties

In this section we are concerned with the calculation of the dipole density time correlation functions and their power spectra. Special attention has been dedicated to the investigation of the origin of the different contributions to the total dipole moment autocorrelation function. A thorough comparison with experimental and dielectric relaxation data, where collective motions are of special interest, has been also performed.

### 4.1. Dipole density time correlations

**Figure 2.**
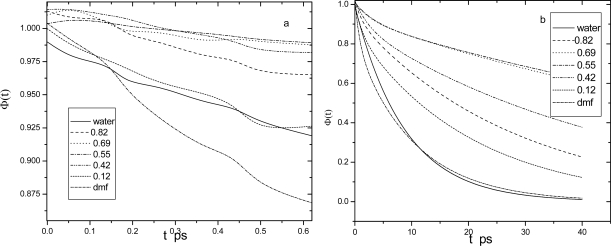
Normalized *k* = 0 dipole density correlations Φ(t) vs time for all simulated systems (b) and details of the short-time behavior (a).

k = 0 Correlations. We begin our discussion of the dynamical dielectric properties with the *k* = 0 normalized lized dipole density time correlation functions Φ(t) for each mixture and for pure DMF, which are depicted in Figure 2.

The curves are labeled according to the DMF mole fraction *x*_D_. We discuss first their short time behavior (Figure 2a). At very early times (from 0 to 0.06 ps), the Φ (t) functions exhibit an inertial decay that is faster for water-richer mixtures because of water’s small moments of inertia. After this initial inertial decay, the functions Φ (t) show fast damped oscillations associated with the H-bonding librational oscillations characteristic of high torque, associating liquids [26].

These librational oscillations are significantly more prominent for water-richer mixtures since water forms on the average four H-bonds, while pure DMF forms none. Such short-time dynamics cannot be very well appreciated from the time-dependent *k* = 0 dipolar relaxation because slow rotational-diffusion processes dominate its decay. Nevertheless, these fast dynamics give rise to prominent, high-frequency peaks in the far-IR spectra, as discussed later on. Figure 2b, on the other hand, reveals the dramatic effect of the addition of water upon the long time dynamics of DMF and vice versa. The results shown in Figure 2b indicate that the dipole density correlations are slowly decaying functions, but their overall decay patterns do not follow a simple composition dependence. It can be seen in Figure 2b that pure DMF and water (solid line) is more relaxed than all mixtures. This interesting behavior correlates well with the fact that the molecular diffusion and long-time single-particle reorientation processes slow upon mixing [8–10,16].

Analysis of the long time (t ≥ 0.6 ps) portions of Φ(t) (Figure 2a), which characterize the slow diffusional relaxation regime of the dipolar correlations, provides dielectric relaxation time parameters that are valuable quantities to be compared against experimental measurements [19]. Associating polar liquids such as alcohols and alcohol-water mixtures usually present multiple time scales in their dielectric relaxation, [26] which are often well described by a sum of exponentials with different time constants, indicating a multiple Debye-like behavior, or by a stretched exponential form, which have also been used to describe the reorientational relaxation in liquids near a glass transition [26] For DMF-water mixtures, we find that either biexponential or stretched exponential functions provide good fits to Φ(t) (Figure 2b) at long times, i.e.;

(14) $$\Phi \left(\text{t}\right)={\text{a}}_{1}\text{exp}\left(-\text{t}/\tau_{1}\right)+{\text{a}}_{2}\text{exp}\left(-\text{t}/\tau_{2}\right)$$

We observe that the relaxation times corresponding to Φ(t) (*k*min,*t*) are smaller than those of Φ(t). The difference is especially significant in the case ofτ_1_. The so-called Debye relaxation time τ_D_ is also reported in Table 3. It is obtained from the following expression:

(15) $$\tau_{\text{D}}\;=\;\underset{\omega \to 0}{\text{lim}}\frac{{ɛ}_{0}-ɛ\left(\omega \right)}{i\omega \left[ɛ\left(\omega \right)-{ɛ}_{\infty }\right]}$$

For a Debye dielectric it should be τ_D_ = τ_1_. According to the results given in Table 3, water-DMF mixtures does not behave like a Debye fluid, since τ_D_ is significantly lower than τ_1_.

Our fitting parameters for each mixture are shown in Table 3. Values of a_1_ ≈ 1 or a_2_ ≈ 0 indicate that the decay is nearly exponential. The results suggest that only at the lowest DMF concentration is the dipolar relaxation more Debye-like, which like in the DMSO-water case, xD = 1.0, F(0) = 1.01 [26].

Comparison with available pure water and DMF experimental data is most conveniently established through the overall main dielectric relaxation time τ*_D_*, which is obtained from integrating Φ(t) with the help of the fitting parameters of Table 3 for all simulated system. The results obtained using the biexponential fits are displayed in Table 3 along with the experimental principal dielectric relaxation time. It can be seen that the simulated dielectric relaxation time τ*_D_* exhibits a nonmonotonic composition dependence, with a maximum around 0. 5 DMF. The overall trend with composition and the magnitude of τ*_D_* for most compositions are in good agreement with the experimental dielectric relaxation time. At *x*_D_ = 0.32 – 0.69, however, the simulations yield a much slower relaxation. A similar effect is also found for the single-particle reorientation time τ_1_ and may be due to the fact that the simulations overestimate the magnitude of molecular interactions around 0.5 composition. The predominance of 2DMF: water stable aggregates around this composition is very likely associated with the dynamical behavior found here. Further work, however, is needed to establish a definitive, more quantitative connection between the formation of these aggregates and the dynamics of the mixtures in the rotational-diffusion regime.

### 4.2. Dielectric relaxation

**Figure 3.**
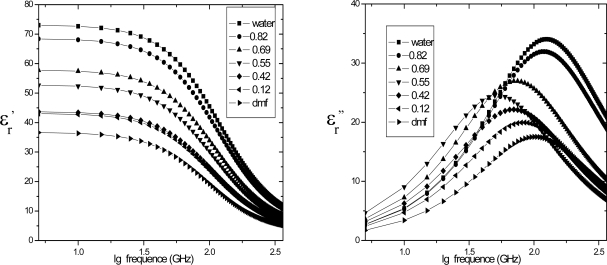
Dielectric constants and losses for mixtures of water and DMF at various concentrations at 298K. The solid line is calculated by Equation 11.

Dielectric constants and losses for DMF-water mixtures at 298 K are shown in Figure 3. A single relaxation peak is observed for the entire concentration range of all the DMF-water mixtures. The peak frequency shifts with increasing DMF concentration, and it reaches a lower frequency for DMF-water mixtures at *x*_D_ = 0.12 – 0.69. This dependency implies that the relaxation observed for the mixtures is due to rotational diffusive motion of both water and DMF molecules. The amplitudes of these high-frequency processes are much lower than that of the primary process. The relaxation times of these high-frequency processes are smaller than that of the primary process. The dielectric relaxation parameters of the primary process strongly depend on the DMF concentration. It is expected that this primary process is due to the cooperative motion of DMF-water molecules through hydrogen bonds. Generally, the primary process observed for various glass-forming polymers, associated liquids, and those water mixtures exhibits an asymmetric loss peak. In a theoretical study, it has been suggested that the motional units in the correlated domains cooperatively move and the heterogeneity of the distribution of the size of the domain results in the asymmetric shape of the loss peak.

## 5. Summary

The static dielectric constant, the dielectric relaxation in frequency of microwave, as well as the microwave spectra of DMF-water mixtures over the whole composition range at room conditions have been investigated from MD simulations. We find good agreement between the simulated and the available experimental dielectric constant for all compositions. Despite the facts that the intermolecular potentials used here are not fully polarizable and that no adjustment of the parameters has been made, the simulated values for ɛ_0_ are only about 10% lower in comparison with the experimental ones. This behavior is, in turn, consistent with the formation of H-bonded aggregates of the type DMF:2 H_2_O, and also with experimental observations. The mixture’s overall dielectric relaxation time τ*_D_* is in good agreement with the experimental principal dielectric relaxation time.

## Figures and Tables

**Table 1.** Intermolecular potential parameters and molecular geometries for water and DMF.

	ɛ/kJmol^−1^	σ/nm	q/e
WATER O	0.6694	0.3120	0
H	0	0	0.2410
Lp	0	0	−0.2410
DMF O_F_	0.210	0.2960	−0.500
C (C = O)	0.105	0.3750	0.500
H_F_ (C = O)	0.015	0.2420	0
N	0.170	0.3250	−0.140
C_M_ (N–CH_3_)	0.066 (0.087)	0.3500 (0.3300)	−0.240
H_M_ (N–CH_3_)	0.030	0.2500	0.060

**Table 2.** System simulation parameters.

DMF	H_2_O	Concentratioṇ mol/l)	ρ/(g/cm^3^) (T = 298K)	L_box_
0	216	0	0.9971	1.8645
30	115	0.2068	0.9959	1.9230
40	84	0.3226	0.9893	1.9529
50	61	0.4505	0.9799	2.0046
60	26	0.6976	0.9615	2.0314
70	12	0.8235	0.9533	2.1092

**Table 3:** Fits to the Normalized Dipole Density Time-Correlation Functions Φ (k = 0, t) in the Postlibrational Regime.

xD	*a*1	τ_1_/ps	*a2*	τ_2_/ps	τ*_D_*/ps	τ*_EXP_*/ps
0	0.99	8.8	0	0	8.2	8.6
0.1266	0.93	28.3	0.09	1.2	15.4	16.2
0.4226	0.93	93.0	0.08	2.6	16.7	18.0
0.5505	0.92	102.0	0.09	3.2	25.1	27.2
0.6976	0.90	46.0	0.12	2.4	23.4	26.2
0.8235	0.87	20.4	0.13	1.3	19.0	22.5
1.0	0.81	10.4	0.21	0.85	9.8	10.2
